# The Performance Evolution of Porous Asphalt Mixtures in Hot In-Place Recycling with the Addition of Different Rejuvenators

**DOI:** 10.3390/ma19122597

**Published:** 2026-06-16

**Authors:** Dongcang Sun, Mingliang Li, Jun Li, Dingding Han, Renfei Li, Yingchen Cui, Wenyue Gao

**Affiliations:** Research Center of Road, Research Institute of Highway Ministry of Transport, Beijing 100088, China; dc.sun@rioh.cn (D.S.); jun.li@rioh.cn (J.L.); dd.han@rioh.cn (D.H.); rf.li@rioh.cn (R.L.); yc.cui@rioh.cn (Y.C.); 18329495027@163.com (W.G.)

**Keywords:** porous asphalt mixture, hot in-place recycling, thermodynamic property, rejuvenator

## Abstract

**Highlights:**

- The performance of asphalt mixtures in HIR was studied with different rejuvenators.
- Bio-based bitumen rejuvenator (B-BBR) reduces asphalt aging, during the HIR.
- B-BBR shows better compatibility and anti-aging performance with asphalt.
- B-BBR enhances the thermal conductivity and low-temperature crack resistance.
- Optimal HIR performance is achieved at 180 °C for 5 min, with 3% B-BBR.

**Abstract:**

With the increased application of porous asphalt, the recycling and reutilization of aged materials have become a critical issue for sustainable pavement engineering. This study investigates the evolution of the performance characteristics of porous asphalt mixtures under high-temperature heating conditions, with the aim of providing a theoretical basis for hot in-place recycling (HIR) technology in the rehabilitation of porous asphalt pavements. The heating states of asphalt, mortar and mixtures in HIR were simulated using controlled oven heating. Their microscopic, mechanical and thermal properties were evaluated under different aging conditions and with the incorporation of different rejuvenators. The results show that asphalt aging intensifies with the increasing heating temperature and time. The incorporation of bio-based rejuvenators significantly alleviates aging effects and demonstrates superior performance compared to conventional rejuvenators. Furthermore, aggregates and rejuvenators enhance the thermal conductivity of materials, while aging reduces the thermal conductivity coefficient and increases the risk of temperature gradient diseases. The rheological properties of asphalt are closely related to the degree of aging. While aging mitigation improves low-temperature cracking resistance and acoustic damping performance, it may compromise high-temperature deformation resistance. In conclusion, to achieve an optimal balance between performance recovery and aging control, it is recommended that the HIR of porous asphalt pavements be conducted at a heating temperature of 180 °C for 5 min, with the addition of 3% bio-based rejuvenator.

## 1. Introduction

Porous asphalt pavement is a typical road surface paved by a porous asphalt (PA) mixture, an environmentally friendly composite material, with a porosity of 18–25% [1,2,3,4]. This unique structure provides multiple functional benefits, including mitigation of traffic noise pollution [5], reduction in pollution and stormwater runoff [6,7,8], and enhanced driving safety under wet conditions due to improved skid resistance [9]. The large air void content of porous asphalt pavement allows surface rainwater to transfer from the structural layer to the base layer [10]. However, prolonged moisture retention within the pavement can accelerate material deterioration, leading to aggregate raveling and structural weakening [11,12,13]. To restore pavement performance, common strategies include rehabilitation, structural overlay, and preventive maintenance. Among these, hot in-place recycling (HIR) has gained increasing attention due to its economic and environmental advantages [14].

HIR technology is a typical recycling method of asphalt pavement [15], which can restore the damaged surface to its original condition [16]. HIR technology is mainly used to treat minor diseases on the surface layer, including cracks, potholes, and rutting [17]. Compared to hot recycling, HIR has significant benefits, including simplified processing, reduced traffic disruption, and improved utilization rate of reclaimed asphalt pavement (RAP). The HIR processes 100% of RAP through heating, milling, adding external materials, hot mixing, paving, and rolling, thereby eliminating the need for material transportation to and from central plants. This substantially reduces energy consumption and greenhouse gas emissions [18]. The high RAP content and performance of recycled asphalt mixture mainly depend on the fusion of blended asphalt [19], which is related to the inherent characteristics of virgin and aged asphalt, recycling agents, mixing time, temperature, etc. [20,21,22]. Yang et al. [23] studied the influence of different factors on the blending efficiency of the aged and virgin asphalt via a micron-Fe_3_O_4_ tracer. The results showed that the blending temperature and time reveal the positive effect on the blending degree. However, during the hot in-place recycling heating process, due to the reaction with oxygen atoms, molecular evaporation and transformation, asphalt will undergo short-term aging. Generally, short-term aging lasts for a short period of time, but the chemical reaction is intense and intensifies with the increase in the high temperature and stirring time [24,25]. Therefore, optimizing the process parameters to balance binder blending and thermal aging is critical.

Different from ordinary asphalt pavement, high-viscosity modified asphalt (HVA) is commonly used as a binder in porous asphalt, particularly in [26,27,28,29]. Due to its complex composition and aging behavior, HVA presents significant challenges for recycling and performance recovery [30]. The HIR of HVA is a complex process, with its core lying in balancing the dual effects of high-temperature aging deterioration and the restorative effect of the regenerant. On the one hand, the performance of high-viscosity aged asphalt itself deteriorates, and it needs to be regenerated by increasing the heating temperature. On the other hand, high-temperature heating will cause new aging. Therefore, the recovery of the performance of asphalt or mixtures is actually the result of the superposition and dynamic balance of high-temperature aging and rejuvenator repair. In addition, due to the need to simultaneously restore drainage function [31,32], raveling resistance and water stability [33,34], porous asphalt pavement faces more stringent requirements for the heating temperature and mixing process. To effectively control the regeneration effect, it is necessary to clearly explain the recovery mechanism and evolution law of the performance of asphalt and its mixtures under the combined action of long-term aging and high-temperature heating.

At present, the types of commonly used rejuvenators for asphalt recycling include aromatic extracts, naphthenic oils, paraffinic oils, tall oils, triglycerides and fatty acids [35]. Abdelaziz et al. [36]. found that all rejuvenators can improve the rheological properties of binder blends, and bio-oil was the most effective. However, bio-oil as a rejuvenator still faces issues such as poor compatibility with asphalt and an unclear long-term aging mechanism. Further research is therefore required.

In this study, a bio-based bitumen rejuvenator previously developed by the research group will be used for HIR of porous asphalt to enhance the compatibility. The long-term aging of asphalt, mortar and mixtures and the heating state during hot in-place recycling were simulated through indoor tests. The microscopic, mechanical and thermal properties of the samples subjected to different aging states and rejuvenator types were systematically characterized to elucidate their performance evolution. The effects of the heating temperature, heating duration, rejuvenator type and dosage on the regeneration performance of porous asphalt mixtures were analyzed.

The research objectives of this study mainly include the following: (1) clarify the variation laws of the thermal and mechanical properties of HVA under high-temperature heating; (2) optimize the HIR process parameters and (3) improve the performance recovery of porous asphalt pavements.

## 2. Materials and Methods

### 2.1. Materials

The high-viscosity additive and styrene–butadiene–styrene (SBS) modified asphalt were used to prepare the HVA. According to Standard Test Methods of Bitumen and Bituminous Mixtures for Highway Engineering (JTG E20-2011 [37]), the properties of the SBS modified asphalt are measured and shown in Table 1. High-viscosity agent was purchased from Nantong Tongsha Asphalt Technology Co., Ltd. (Nantong, China), and its properties were shown in Table 2 (JT T860.2-2013 [38]). Mineral powder was supplied by Zaozhuang Taierzhuang District Xinding Calcium Carbonate Factory (Zaozhuang, China). Basalt was produced in Chifeng, Inner Mongolia (China). Bitumen rejuvenator was supplied by XiYueFa Group (Taiyuan, China).

**Figure 1:**
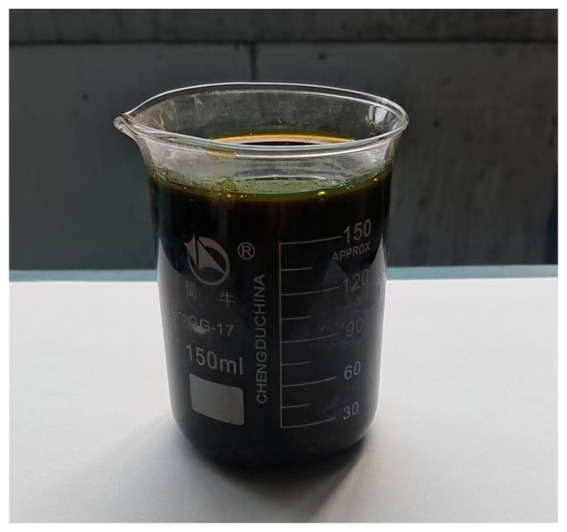
The photograph of the bio-based bitumen rejuvenator.

To further enhance the antioxidant performance, a bio-based bitumen rejuvenator (B-BBR) (Figure 1) independently developed by the research group was employed. Its main components include vegetable oil (47–55 wt%), terpene phenolic resin (12–15 wt%), bio-based modifier (8–12 wt%), and other additives such as antioxidants and surfactants. This bio-based modifier incorporates algae extracts, chitin and sulfonated lignin, which enhance the antioxidant capacity, bonding performance, and low-temperature flexibility. The components of the algae extract include brown algae, chlorella and spirulina. The petroleum-based rejuvenator was typically formulated with a base oil such as aromatic oil or furfural extract oil, a resin like C9 petroleum resin, and some functional additives. Different from conventional petroleum-based rejuvenators that simply soften aged asphalt, bio-based asphalt rejuvenators restore and replenish the missing components in aged asphalt through chemical reactions, thereby rebalancing its original chemical composition and structure and effectively restoring the performance of the aged asphalt. The bio-based formulation offers improved environmental sustainability by reducing dependence on non-renewable resources and minimizing secondary pollution. Additionally, its biodegradable nature further supports environmentally friendly pavement technologies. The performances of B-BBR were measured, according to Standard Test Methods of Bitumen and Bituminous Mixtures for Highway Engineering (JTG E20-2011), as shown in Table 3.

### 2.2. Preparation of the HVA, Mortar and Mixtures

HVA was prepared through a controlled heating, blending, and shearing process. Firstly, the asphalt was heated in an oven at 160–170 °C for 3–4 h until completely melted. Secondly, the asphalt was transferred to an induction cooker at 180 °C and the high-viscosity agent was added in three portions while stirring at a rate of 200–300 r/min. The time interval between each addition should be 2 min. Thirdly, a shearing machine was used to shear asphalt at 180 °C for 30 min. Finally, the asphalt was placed in an oven at 180 °C and maintained for 30 min to obtain the HVA. Additionally, the mass ratio of asphalt to high-viscosity agent in HVA was 92:8. According to Standard Test Methods of Bitumen and Bituminous Mixtures for Highway Engineering (JTG E20-2011), the properties of HVA are presented in Table 4.

**Figure 2:**
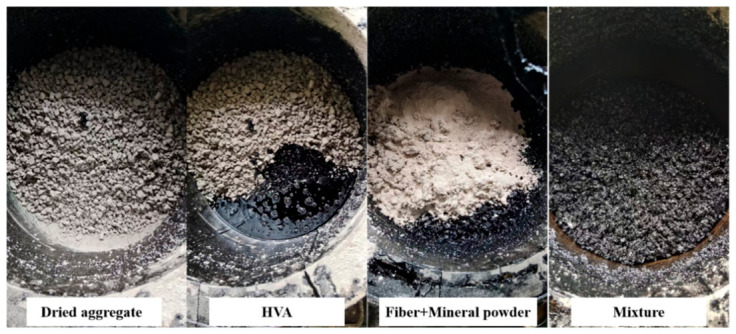
The preparation of the mixture.

The particle size of the mineral powder used for preparing the mortar was 0–0.3 mm, and the mass ratio of HVA to mineral powder was 1:1. The preparation procedure for the asphalt mortar was similar to that of HVA, except that HVA was used as the binder and mineral powder was added instead of the high-viscosity additive. The mixtures were prepared according to Chinese Transportation industrial specification Technical Specifications for Design and Construction of Porous Asphalt Pavement (JTG/T 3350-03-2020 [40]), as shown in Figure 2. The porous asphalt mixture followed a PAC-13 gradation with an air void content of 20%. In addition, basalt fibers were incorporated at 0.1% by total mixture weight to enhance the structural stability and raveling resistance.

### 2.3. Sample Fabrication

According to Standard Test Methods of Bitumen and Bituminous Mixtures for Highway Engineering (JTG E20-2011), the asphalt and mortar were aged by using a thin-film oven test (T0609-2011), heated for 5 h at 163 °C. The aged mixture was subsequently prepared by the aged asphalt and mortar. Then, short-term aging was performed in a high-temperature oven under the conditions specified in Table 5 to replicate the thermal environment during hot in-place recycling. Among them, samples H7–H9, M6–M13, P2 and P4 were added with a certain proportion of rejuvenator before short-term aging. Specifically, in a 100 mL beaker, the asphalt or mortar (50 g) were heated to the corresponding temperature. After adding the rejuvenator and stirring, the temperature was kept constant until the corresponding time. The mixture was made by heating the aged asphalt or mortar to the corresponding temperature, then adding rejuvenator, aggregates, etc., and mixing them for the corresponding time to form a Marshall test piece. Based on engineering experience, the dosage of bitumen rejuvenator was selected at 1%, 3%, and 5%. After long-term aging, porous asphalt mixtures containing HVA or asphalt mortar were compacted into small Marshall specimens for subsequent testing. For comparison, unaged HVA was designated as sample H0 and used as a reference material.

### 2.4. Test Methods

#### 2.4.1. Aging Index Test

The attenuated total reflectance method (ATR, Thermo Nicolet iS5 spectrometer; Thermo Fisher Scientific: Wilmington, DE, USA) was used to characterize the functional groups on the surface of the samples to calculate the aging index of the HVA. Each sample was measured three times and the average value was taken.

#### 2.4.2. Microscopic Morphology Test

The microscopic morphology of the samples was studied by Leica DMI 3000 B fluorescence microscope (Leica Microsystems (Shanghai) Trading Co., Ltd.: Shanghai, China). The wavelength range of the excitation light used in the test is 340 nm–390 nm, 460 nm–495 nm and 530 nm–550 nm, with an amplification factor of 100 times.

#### 2.4.3. Thermal Conductivity Test

**Figure 3:**
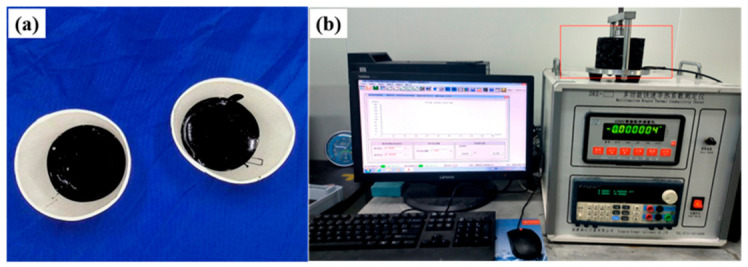
(**a**) The HVA samples of the thermal conductivity texts, and (**b**) the thermal conductivity tester with a mixture sample.

A DRE-III Multifunction rapid Thermal Conductivity Tester was used to characterize the thermal conductivity of the samples. Each sample was measured three times and the average value was taken. When the test temperature was ≤80 °C, 50 g of asphalt or mortar was placed in a paper cup to form a thermal conductivity test sample, as shown in Figure 3a. When the test temperature was 200 and 300 °C, the test probe was inserted into 50 g of molten asphalt or mortar for testing. The thermal conductivity of the mixture was tested using two small Marshall specimens, as shown in Figure 3b.

#### 2.4.4. Rheological Property Test

**Figure 4:**
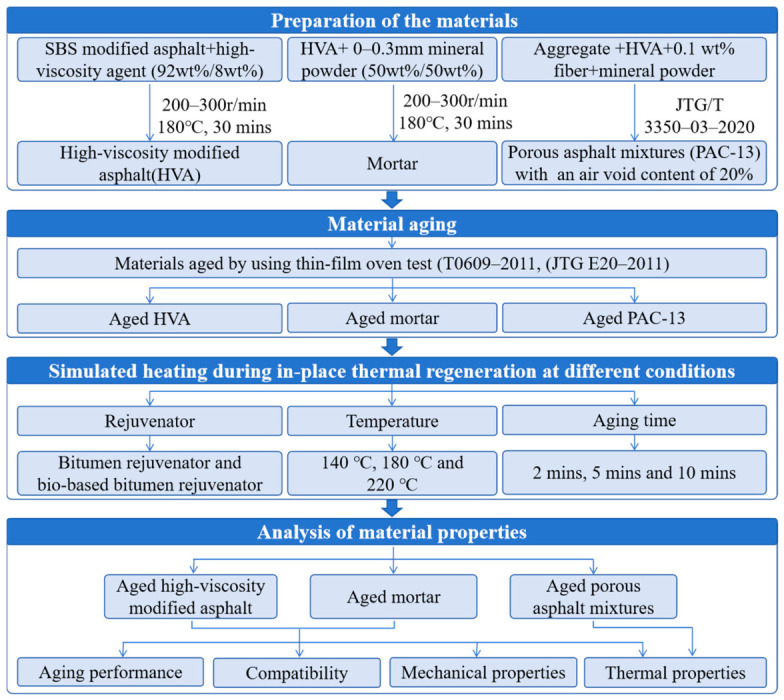
The flow chart of the experiment design.

The rheological properties of the samples were evaluated using a Dynamic Shear Rheometer and a low-temperature Beam Bending Rheometer. The shear rate scanning tests on the samples were performed on a TA DHR-3 Dynamic Shear Rheometer (Waters^TM^ (Shanghai) Co., Ltd.: Shanghai, China). The test temperature was set to 46–124 °C, with a sweep frequency of 10 rad/s and a strain level of 10%. The bend creep stiffness was characterized by a CANNON TE Beam Bending Rheometer (Earth Products China Limited: Hongkong, China) at a test temperature of −18 °C. The centralized loading time was set at 240 s, and the stiffness modulus and creep rates at 8 s, 15 s, 30 s, 60 s, 120 s, and 240 s were obtained. Each sample was measured three times. Figure 4 shows the flow chart of the experiment design.

## 3. Results and Discussion

### 3.1. Aging Index of Samples

**Figure 5:**
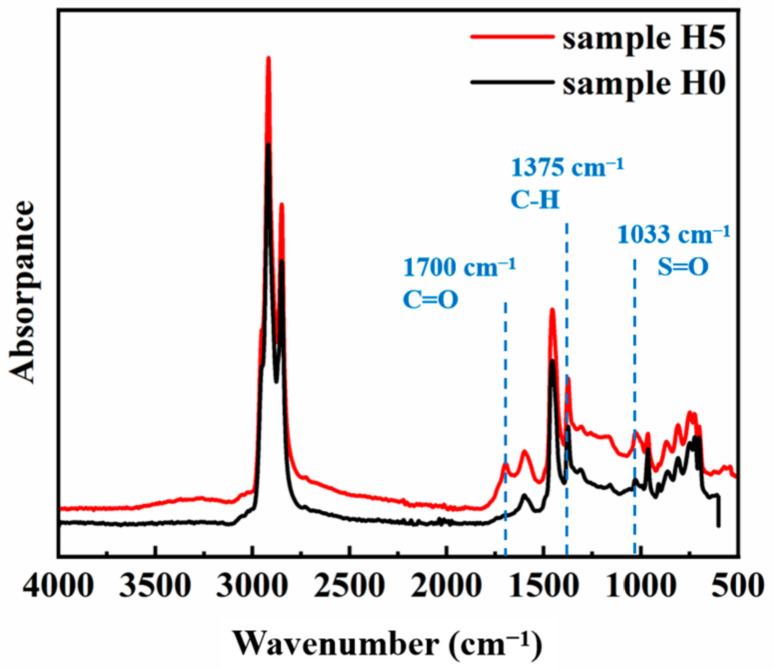
FTIR-ATR spectrum of sample H0 and H5.

The aging index (AI) of the asphalt was calculated according to the FTIR-ATR spectrum of the samples. Representative spectra of the unaged HVA (H0) and aged sample H5 are presented in Figure 5. Compared to the unaged HVA, the new peaks at 1033 cm^−1^ and 1700 cm^−1^ belong to the stretching vibration of C=O and S=O, which proves the aging of the asphalt. The absorption peak at 1375 cm^−1^ is assigned to C-H.

The quantitative aging analysis was conducted by using the area ratios of the carbonyl and sulfoxide groups to the saturated C-H functional groups respectively. The two ratios were respectively called the carbonyl coefficient (CI) and the sulfoxide coefficient (SI), and the sum of CI and SI was called the aging coefficient AI [41,42]. The calculation equations are as follows:

CI = A_C=O_/A_C-H_ (1)

SI = A_S=O_/A_C-H_ (2)

AI = CI + SI(3)

where A_C=O_ is the absorption peak area of the C=O bond at 1700 cm^−1^, A_S=O_ is the absorption peak area of the S=O bond at 1033 cm^−1^, and A_C-H_ is the absorption peak area of the C-H bond at 1375 cm^−1^. The AIs of the samples were calculated, as shown in Table 6. It can be seen that as the heating time and temperature increase, the AI gradually increases. Adding a bitumen rejuvenator can reduce the aging degree of asphalt, and as the amount of bitumen rejuvenator added increases, the aging degree of asphalt decreases. The AI of aged HVA can be reduced from 0.29 to 0.15 at most by HIR with the addition of B-BBR. Compared with bitumen rejuvenator, B-BBR has a better recovery effect on aged asphalt.

### 3.2. Fluorescent Microscope Analysis

**Figure 6:**
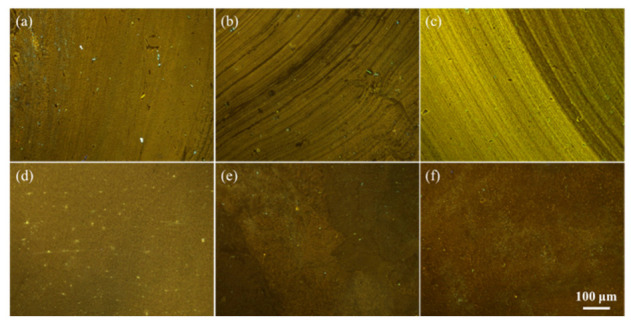
Fluorescence images of (**a**) H2; (**b**) H3; (**c**) H4; (**d**) H7; (**e**) H8 and (**f**) H9.

The fluorescence images of HVA under different heating temperatures magnified by 100 times are shown in Figure 6. Among these, samples H7–H9 were modified with 3% B-BBR. Under ultraviolet excitation, the polymer-rich phase in HVA exhibits bright yellow fluorescence, whereas the asphalt matrix appears dark due to the absence of fluorescence response. As can be seen from Figure 6, after the addition of the rejuvenator, the organic phase was distributed in the asphalt in the form of spherical particles. As the B-BBR dosage increased, the particle size of the organic phase decreased significantly, and the particle uniformity improved. This indicated that the B-BBR has good compatibility with HVA, and its addition does not cause the aggregation of polymer.

**Figure 7:**
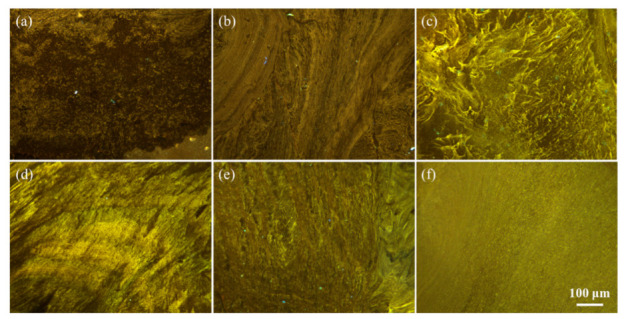
Fluorescence images of (**a**) M1; (**b**) M2; (**c**) M6; (**d**) M7; (**e**) M8 and (**f**) M9.

Figure 7 shows the fluorescence diagrams of asphalt mortar with different types and contents of bitumen rejuvenator. The addition of common bitumen rejuvenator leads to slight aggregation of the polymer phase in HVA, while B-BBR shows good compatibility. Moreover, as the amount of B-BBR added increases, the polymer phase is dispersed more evenly.

### 3.3. High-Temperature Thermal Conductivity

In asphalt pavement, thermal conductivity governs the rate of heat transfer within the pavement structure. Materials with higher thermal conductivity enable faster heat dissipation from warmer to cooler regions, thereby reducing the magnitude of temperature gradients. Lower-temperature gradients, in turn, decrease thermal stress accumulation, which is a primary cause of low-temperature cracking and other temperature-related distresses [43,44]. Table 7 presents the thermal conductivity coefficients of HVA, mortar and mixture at different temperatures. To further clarify the heat transfer performance of porous asphalt pavement during hot in-place recycling, the thermal conductivity coefficients of HVA and mortar at 200 °C and 300 °C were also measured.

Compared with the unaged H0, the thermal conductivity of the aged H1 decreased significantly, indicating that oxidative aging adversely affects the heat transfer capacity of asphalt. At a given temperature, the thermal conductivity follows the order: HVA < mortar < mixture. This trend is primarily governed by differences in material composition and structure. Furthermore, with the addition of B-BBR, the thermal conductivity of HVA, mortar and mixture increases, indicating an improvement in thermal conductivity, which is related to the reduction in the aging degree of asphalt. Below 80 °C, as the temperature rises, the thermal conductivity of the samples gradually increases, indicating that a low-temperature environment can affect the thermal conductivity of porous asphalt pavement and increase the occurrence of diseases caused by temperature gradients. After HIR of the porous asphalt mixtures under the condition of 180 °C heating temperature for 5 min, together with 3% B-BBR, its thermal conductivity coefficient at 80 °C is 0.787 W/(m·K). However, at elevated temperatures (200 °C and 300 °C), a decreasing trend in thermal conductivity is observed with a further temperature increase. Excessively high hot in-place recycling temperatures can cause asphalt to age and affect its thermal conductivity.

### 3.4. Low-Temperature Rheological Performance

**Figure 8:**
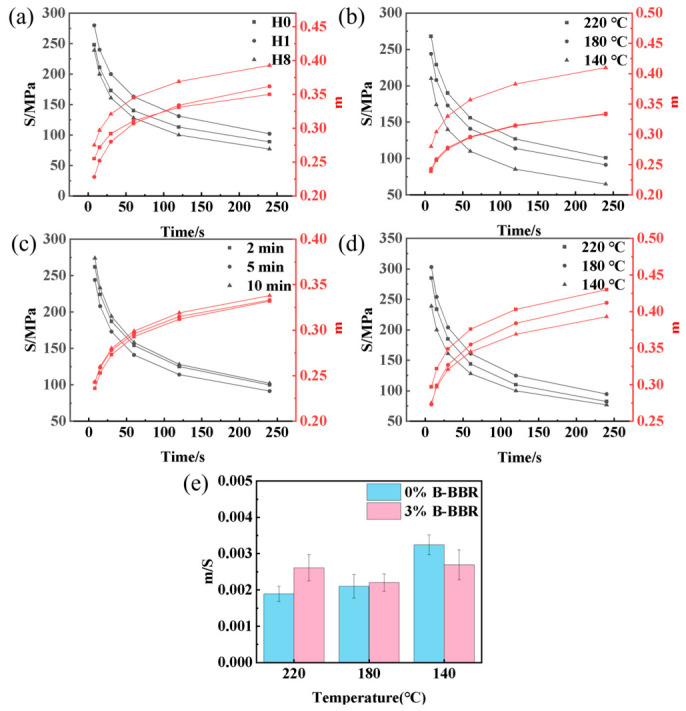
(**a**) S, m values of the unaged HVA, aged HVA and HVA after hot regeneration with 3% B-BBR; and S, m values of the HVA at (**b**) different heating temperatures with a heating time of 5 min and 0% B-BBR; (**c**) different heating time with a heating temperature of 180 °C and 0% B-BBR; (**d**) different heating temperatures with a heating time of 5 min and 3% B-BBR; and (**e**) m/S values of the HVA at different heating temperatures with 0% and 3% B-BBR.

The Beam Bending Rheometer test results of unaged HVA, aged HVA and HVA after hot regeneration with B-BBR under different heating temperatures and time conditions are shown in Figure 8.

The Beam Bending Rheometer test was conducted at −18 °C, and the creep rate (m) and stiffness modulus (S) were used as evaluation indicators, which were traditionally used to evaluate the deformation and flow behavior of materials under stress. As can be seen from Figure 8a, after asphalt ages, the S value increases and the m value decreases, indicating that the low-temperature crack resistance of HVA gradually declines as it ages. After regenerating aged asphalt with B-BBR, its low-temperature rheological performance has significantly improved and is superior to that of new asphalt. This is mainly because the rejuvenator’s active components physically soften the material by replenishing lost light fractions, while simultaneously chemically breaking down harmful polar aggregates and partially repairing the SBS network, together achieving a more uniform and optimized microstructure than the original.

During the asphalt recycling process, increasing the heating temperature and duration leads to a gradual increase in S and a decrease in the m value (Figure 8b,c), indicating that excessive thermal exposure exacerbates aging and weakens low-temperature performance. Figure 8d shows the variation in S and m values after incorporating 3% bio-based rejuvenator prior to heating. Compared with the absence of B-BBR, both the S and m values of HVA increased. A single S and m value cannot comprehensively evaluate the low-temperature rheological performance of HVA. Therefore, the index m/S was further studied when the test time was 60 s, as shown in Figure 8e. At heating temperatures of 180 and 220 °C, the addition of B-BBR significantly increased m/S, indicating an improvement in low-temperature performance. At a lower heating temperature of 140 °C, the rejuvenator exhibits limited effectiveness due to insufficient interaction with the asphalt matrix, resulting in a reduction in performance.

**Figure 9:**
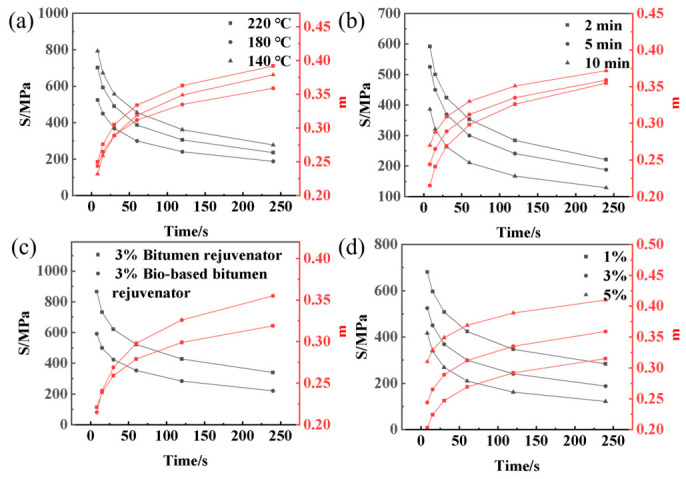
S, m values of the mortar: (**a**) at different heating temperatures with a heating time of 5 min and 3% B-BBR; (**b**) at different heating time with a heating temperature of 180 °C and 3% B-BBR; (**c**) with different bitumen rejuvenator at a heating temperature of 180 °C for 5 min; (**d**) with different amounts of B-BBR at a heating temperature of 180 °C for 5 min.

The S and m values of mortar are shown in Figure 9. The effects of different heating temperatures and times, as well as the types and dosages of rejuvenator on the low-temperature rheological properties of the mortar, were studied. As can be seen from Figure 9a,b, with the increase in the heating temperature and time, the S value of the mortar gradually increases and the m value gradually decreases. Compared with ordinary bitumen rejuvenator, B-BBR is more conducive to improving the value of m and decreasing S (Figure 9c). Moreover, as the addition amount of B-BBR increases, S gradually decreases and m increases (Figure 9c), illustrating an improvement in the low-temperature crack resistance of the mortar.

### 3.5. High-Temperature Rheological Performance

**Figure 10:**
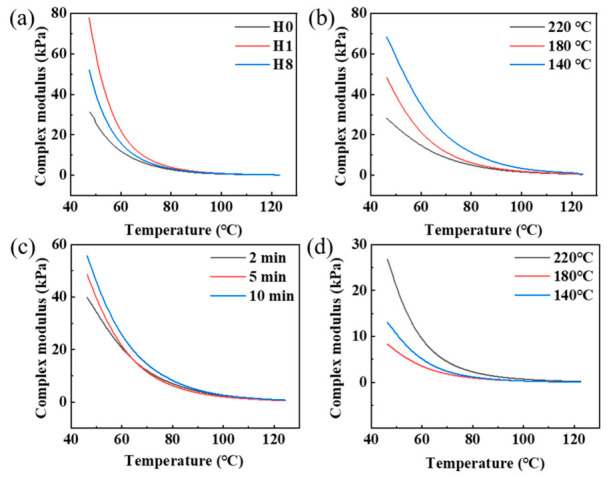
(**a**) Complex modulus of unaged HVA, aged HVA and HVA after hot regeneration with 3% B-BBR; and complex modulus of the HVA at (**b**) different heating temperatures with a heating time of 5 min and 0% B-BBR; (**c**) different heating time with a heating temperature of 180 °C and 0% B-BBR; (**d**) different heating temperatures with a heating time of 5 min and 3% B-BBR.

Figure 10 presents the test results of the high-temperature rheological performance of asphalt during heating. Figure 10a shows the complex modulus of unaged HVA, aged HVA and HVA after hot regeneration with B-BBR. Asphalt aging leads to an increase in the complex modulus. After asphalt is regenerated with bio-based rejuvenator, the complex modulus decreases, but it is still greater than that of unaged asphalt. As can be seen from Figure 9c and Figure 10b, the complex modulus of HVA increases with the rise in the heating temperature and time. Adding 3% B-BBR to the HVA before heating can significantly reduce its complex modulus, confirming the recovery effect of the B-BBR on the aging of the HVA. At a heating temperature of 180 °C, the complex modulus at 46 °C decreases from 48.4 kPa (without rejuvenator) to 8.3 kPa (with rejuvenator), representing a substantial reduction in stiffness.

**Figure 11:**
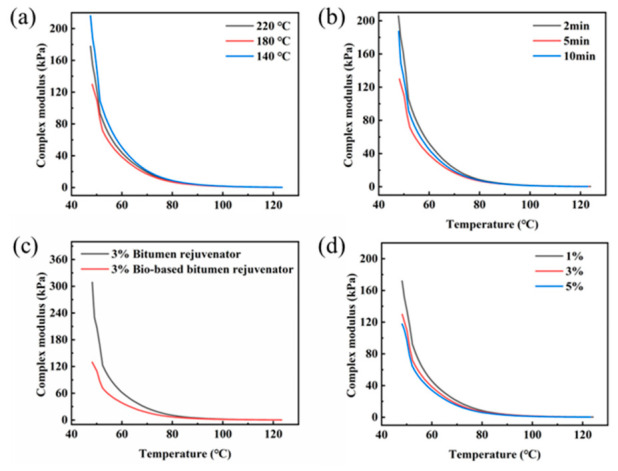
Complex modulus of the mortar: (**a**) at different heating temperatures with 3% B-BBR; (**b**) at different heating time with 3% B-BBR; (**c**) with different bitumen rejuvenator; (**d**) with different amounts of B-BBR.

**Figure 12:**
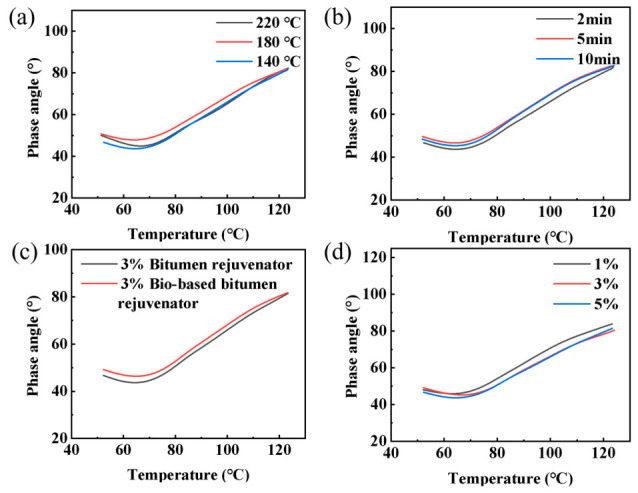
Phase angle of the mortar: (**a**) at different heating temperatures with 3% B-BBR; (**b**) at different heating time with 3% B-BBR; (**c**) with different bitumen rejuvenator; (**d**) with different amounts of B-BBR.

Figure 11 and Figure 12 respectively show the complex modulus and phase angle of asphalt mortar under different heating conditions. As the temperature rises, the complex modulus gradually decreases, but the phase angles show a trend of first decreasing and then increasing. A higher complex modulus and a lower phase angle correspond to better resistance to high-temperature deformation. As shown in Figure 11a,b and Figure 12a,b, at 180 °C and heated for 5 min, the mortar has the lowest complex modulus and the largest phase angle. This is because under this condition, the aging coefficient of the mortar is the smallest, and the aging of asphalt will enhance its high-temperature anti-deformation ability. Similarly, compared with the addition of common regenerants, the complex modulus of the glue paste with bio-based regenerants is smaller and the phase angle is larger. Moreover, as the amount of biological regenerant added increases, the complex modulus gradually decreases and the phase angle gradually increases.

## 4. Conclusions

In summary, by simulating the heating states of HVA, mortar and mixture in the hot in-place recycling process of porous asphalt pavement, the aging degree, microstructure, thermal conductivity, high-temperature and low-temperature rheological properties of the samples under different conditions were studied. The main conclusions are summarized as follows:

(1) The aging degree of asphalt increases with a higher heating temperature and prolonged heating time. The addition of B-BBR can significantly reduce the aging degree of asphalt. The AI of aged HVA can be reduced from 0.29 to 0.15 at most by HIR with the addition of B-BBR. Compared with ordinary bitumen rejuvenator, B-BBR demonstrates superior performance in reducing the aging degree of asphalt and exhibits better compatibility with asphalt.

(2) The addition of aggregates and rejuvenator increases the thermal conductivity of all the HVA, mortar and mixture. After HIR of the porous asphalt mixtures under the condition of 180 °C heating temperature for 5 min, together with 3% B-BBR, its thermal conductivity coefficient at 80 °C is 0.787 W/(m·K).

(3) A lower aging degree improves the low-temperature crack resistance of the HVA and mortar but reduces the high-temperature deformation resistance. However, as the degree of aging decreases and the phase angle increases, it indicates that asphalt has a better damping effect, which can effectively reduce road surface vibration and noise and improve driving comfort.

(4) Taking into account the aging of hot in-place recycled asphalt under heating conditions and the interaction between the regenerant and asphalt, an optimal condition of 180 °C heating temperature for 5 min, together with 3% B-BBR, is recommended to achieve a balanced improvement in the performance of recycled porous asphalt pavement.

(5) Due to the limitations of the experimental conditions and time, this study still has some limitations and work to be carried out. In the subsequent research, we will further enhance the authenticity of the HIR heating simulation and supplement the tests of the mixture such as the Cantabro loss, permeability, moisture susceptibility, rutting resistance, and raveling resistance. In addition, further tests on the durability, drainage function and on-site workability will be conducted.

## Figures and Tables

**Table 1:** The properties of the SBS modified asphalt.

Test Items	Test Results	Unit	Test Method
Penetration (25 °C, 100 g, 5 s)	62.0	0.1 mm	T 0604-2011
Softening point (TR&B)	90.8	°C	T 0606-2011
Ductility (5 °C, 5 cm/min)	35.0	cm	T 0605-2011
Solubility	99.8	%	T 0607-2011
Elastic recovery (25 °C)	93	%	T 0662-2000
Brookfield viscosity (135 °C)	2.362	Pa·s	T 0625-2011
Density (25 °C)	1.031	g/cm^3^	T 0603-2011
Residue after TFOT			
Quality change	0.018	%	T 0609-2011
Penetration ratio (25 °C, 100 g, 5 s)	76.5	%	T 0604-2011
Ductility (5 °C, 5 cm/min)	22	cm	T 0605-2011

**Table 2:** The properties of high-viscosity agent.

Test Items	Test Results	Unit	Test Method
Appearance	Graininess, uniform and plump	-	JT T860.2-2013
Mass of a single particle	0.022	g	JT T860.2-2013
Density	0.983	g/cm^3^	JT T860.2-2013
Melt index	8.6	g/10 min	JT T860.2-2013

**Table 3:** The performances of B-BBR.

Test Items	Test Results	Unit	Test Method
Brookfield viscosity (65 °C)	0.034	Pa·s	T 0625-2011
Flash point	>310	°C	T 0611-2011
Marcelene content	91.7939	%	SN/T 3118-2012 [39]
Density (15 °C)	0.926	g/cm^3^	T 0603-2011
Residue after TFOT			
Viscosity ratio	0.91	-	T 0619-2011
Quality change	0.09	-	T 0610-2011

**Table 4:** The properties of HVA.

Test Items	Test Results	Unit	Test Method
Penetration (25 °C, 100 g, 5 s)	44.5	0.1 mm	T 0604-2011
Softening point (TR&B)	96.0	°C	T 0606-2011
Ductility (5 °C, 5 cm/min)	44.0	cm	T 0605-2011
Dynamic viscosity (60 °C)	552,960	Pa·s	T 0620-2011
Brookfield viscosity (170 °C)	1.102	Pa·s	T 0625-2011
Solubility	99.6	%	T 0607-2011
Elastic recovery (25 °C)	96.5	%	T 0662-2000
Relative density (25 °C)	1.036	-	T 0603-2011
Residue after TFOT			
Quality change	0.536	%	T 0609-2011
Penetration ratio (25 °C, 100 g, 5 s)	83.5	%	T 0604-2011
Ductility (5 °C, 5 cm/min)	29	cm	T 0605-2011

**Table 5:** The short-term aging conditions of the samples.

Sample	Composition	Rejuvenator		Temperature /°C	Aging Time /min
		Bitumen Rejuvenator	Bio-Based Bitumen Rejuvenator		
H1	HVA	-	-	-	-
H2	HVA	-	-	220	5
H3	HVA	-	-	180	5
H4	HVA	-	-	140	5
H5	HVA	-	-	180	2
H6	HVA	-	-	180	10
H7	HVA	-	3%	220	5
H8	HVA	-	3%	180	5
H9	HVA	-	3%	140	5
M1	mortar	-	-	220	5
M2	mortar	-	-	180	5
M3	mortar	-	-	140	5
M4	mortar	-	-	180	2
M5	mortar	-	-	180	10
M6	mortar	3%	-	180	5
M7	mortar	-	1%	180	5
M8	mortar	-	3%	180	5
M9	mortar	-	5%	180	5
M10	mortar	-	3%	220	5
M11	mortar	-	3%	140	5
M12	mortar	-	3%	180	2
M13	mortar	-	3%	180	10
P1	porous asphalt mixtures (HVA)	-	-	180	5
P2	porous asphalt mixtures (HVA)	-	3%	180	5
P3	porous asphalt mixtures (mortar)	-	-	180	5
P4	porous asphalt mixtures (mortar)	-	3%	180	5

**Table 6:** The AI of the samples.

Sample	A_C-H_	A_C=O_	A_S=O_	CI	SI	AI
H1	0.773	0.119	0.102	0.154	0.132	0.29
H2	0.903	0.262	0.229	0.290	0.254	0.54
H3	0.985	0.093	0.205	0.094	0.208	0.30
H4	0.508	0.033	0.069	0.065	0.136	0.20
H5	0.797	0.058	0.165	0.073	0.207	0.28
H6	0.708	0.069	0.314	0.097	0.444	0.54
H7	0.817	0.05	0.252	0.061	0.308	0.37
H8	0.708	0.027	0.101	0.038	0.143	0.18
H9	0.838	0.050	0.075	0.060	0.089	0.15
M1	0.783	0.195	0.471	0.249	0.602	0.85
M2	0.740	0.163	0.192	0.220	0.259	0.48
M3	0.718	0.134	0.163	0.187	0.227	0.41
M4	0.720	0.084	0.164	0.117	0.228	0.34
M5	0.691	0.135	0.234	0.195	0.339	0.53
M6	0.712	0.066	0.143	0.093	0.201	0.29
M7	0.663	0.053	0.249	0.080	0.376	0.46
M8	0.658	0.047	0.103	0.071	0.157	0.23
M9	0.777	0.039	0.105	0.050	0.135	0.19
M10	0.648	0.037	0.210	0.057	0.324	0.38
M11	0.732	0.039	0.108	0.053	0.148	0.20
M12	0.694	0.051	0.102	0.073	0.147	0.22
M13	0.442	0.073	0.133	0.165	0.301	0.47

**Table 7:** The thermal conductivity coefficients of samples.

Sample	Thermal Conductivity Coefficient (W/(m·K))				
	20 °C	50 °C	80 °C	200 °C	300 °C
H0	0.171	0.195	0.266	0.223	0.196
H1	0.163	0.185	0.209	0.210	0.186
H3	0.155	0.179	0.203	0.181	0.168
H8	0.159	0.182	0.213	0.205	0.190
M2	0.162	0.198	0.214	0.201	0.185
M8	0.182	0.205	0.244	0.208	0.187
P1	0.650	0.674	0.702	-	-
P2	0.660	0.682	0.710	-	-
P3	0.572	0.611	0.647	-	-
P4	0.736	0.760	0.787	-	-

## Data Availability

The original contributions presented in this study are included in the article. Further inquiries can be directed to the corresponding author.

## Abbreviations

The following abbreviations are used in this manuscript:

AI	aging index
B-BBR	bio-based bitumen rejuvenator
CI	carbonyl coefficient
HIR	hot in-place recycling
HVA	high-viscosity modified asphalt
PA	porous asphalt
RAP	reclaimed asphalt pavement
SBS	styrene–butadiene–styrene
SI	sulfoxide coefficient
